# Integrated Ultrasonic Platform for Bioelectronic Control through Biological Barriers Based on Metasurface

**DOI:** 10.1002/advs.75563

**Published:** 2026-05-07

**Authors:** Chuanxin Zhang, Hanjie Xiao, Xue Jiang, Dean Ta

**Affiliations:** ^1^ College of Future Information Fudan University Shanghai China; ^2^ State Key Laboratory of Integrated Chips and System Fudan University Shanghai China

## Abstract

Closed‐loop bioelectronic systems that adapt stimulation to real‐time physiological feedback hold transformative potential for treating neurological and cardiac disorders and are emerging as key components of future ultrasonic brain–machine interfaces (uBMIs). Realizing this requires the simultaneous achievement of millimeter‑scale deep‐tissue targeting, artifact‐free physiological feedback, and robust wireless power and data transfer, which remain elusive with current methods. Here, we present an integrated ultrasonic platform engineered to overcome these fundamental limitations. We propose a physics‐constrained metasurface design framework to enable high‐resolution multifocal ultrasound energy delivery through highly aberrating biological barriers such as the skull and ribs, achieving improved experimental targeting accuracy (e.g., ±6.5% intensity uniformity across multiple foci). We demonstrate the platform's adaptive stimulation capabilities through two distinct paradigms: attention‐based ultrasound stimulation and cardiac‐synchronized ultrasound stimulation. Furthermore, we introduce a novel dual‐channel acoustic link that enables continuous wireless power and wireless data streaming through the skull with a single acoustic metasurface, demonstrating robustness even with a 400‐fold power differential. This integrated ultrasonic framework, providing seamless integration of precise spatial targeting through biological barriers, adaptive physiological feedback, and untethered operation, contributes to the development of next‐generation uBMIs and closed‐loop bioelectronic therapies.

## Graphical abstract

A metasurface‐enabled ultrasonic platform achieves precise multi‐focal control through biological barriers, real‐time physiology‐driven modulation, and simultaneous wireless power and data transmission. By unifying wavefront engineering, closed‐loop feedback, and acoustic communication, this work establishes key enabling technologies toward scalable ultrasonic brain‐machine interfaces in complex tissue environments.

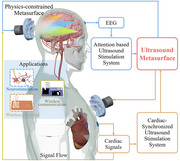

## 1 Introduction

The intricate interplay of electrical signals within the human brain and heart governs fundamental processes such as cognition, emotion, and physiological stability. Non‐invasive monitoring techniques, such as electroencephalography (EEG) for brain activity and electrocardiography (ECG) for cardiac rhythms, offer real‐time insights into these dynamic physiological states. The ability not only to monitor but also dynamically modulate these signals in a closed‐loop manner, where therapeutic stimulation adapts to physiological feedback, holds potential for targeted interventions and enhanced therapeutic efficacy beyond traditional open‐loop approaches [1, 2]. However, the realization of effective closed‐loop bioelectronic interfaces has been fundamentally limited by the inherent constraints of conventional electromagnetic (EM) technologies. Whether using non‐invasive methods like transcranial direct current stimulation or implantable systems such as Deep Brain Stimulation (DBS), these techniques generate strong electromagnetic fields that interfere with concurrently recorded physiological signals [3, 4]. This signal contamination compromises the integrity of the feedback loop necessary for real‐time adaptive control. This conundrum is particularly acute in adaptive DBS, where the most effective biomarkers for closed‐loop control are often rendered unusable by these artifacts, critically impeding the development of next‐generation therapies [5, 6]. Compounding this signal integrity problem, the fundamental physics of EM wave propagation in tissue presents a dual challenge for spatial control and wireless operation. Substantial attenuation at higher frequencies not only fundamentally limits the achievable spatial resolution and deep‐tissue penetration for stimulation, but also severely hampers reliable wireless power transfer for energy‐intensive implants [7, 8]. This creates a difficult trade‐off between high battery consumption from continuous operation and inefficient, potentially unsafe wireless recharging due to tissue heating and electromagnetic interference.

Ultrasound has emerged as a compelling alternative modality for bioelectronic interfaces, offering distinct advantages in deep tissue penetration, high spatial resolution, and compatibility with real‐time physiological monitoring [9, 10, 11]. Unlike electromagnetic stimulation, ultrasound operates through mechanical waves, inherently eliminating electromagnetic artifacts that plague concurrent EEG or ECG recordings [12, 13]. Low‐intensity focused ultrasound [14] has demonstrated the capacity to modulate neural activity in preclinical models with high precision and minimal invasiveness, establishing ultrasound neuromodulation (UNM) [15] as a promising avenue for both fundamental neuroscience research and targeted therapeutic intervention [16]. Moreover, the mechanical nature of ultrasound lends itself well to wireless applications, enabling efficient power transfer and data transmission through biological tissue with reduced interference, which are essential for the effective closed‐loop bioelectronic interfaces [17, 18, 19].

Despite these advantages, a fundamental challenge hindering the implementation of ultrasound in closed‐loop systems lies in achieving precise acoustic wavefront shaping through complex and heterogeneous biological tissue, particularly thick structures like the human skull and ribcage. While promising UNM studies have been conducted in small animal models [20, 21] (whose thinner skulls present a less formidable acoustic barrier), the thick and acoustically heterogeneous structure of the human skull induces significant acoustic distortions that scatter the wavefront and degrade focal accuracy [22, 23]. Recent works exploring impedance matching or resonance principles [24, 25, 26, 27] have shown improved ultrasound transmittance through such barriers, but these methods primarily demonstrate single‐point ultrasound focusing. They lack the capability for complex field manipulation necessary for the adaptive, multifocal control required by the closed‐loop bioelectronic system. Acoustic metasurfaces provide a powerful route for advanced wavefront shaping [28, 29], in which holographic principles are typically used to derive the phase modulation required for a prescribed pressure field [30, 31, 32, 33], and the metasurface is then designed to realize that phase profile [34, 35, 36]. However, this two‐step method is insufficient for accomplishing the complex and precise ultrasound control through the biological barriers, due to the simplified acoustic models that neglect nonlocal interactions and ultrasound diffraction within the metasurface and the barrier [37]. Ultimately, integrating ultrasound into a comprehensive closed‐loop bioelectronic control platform necessitates more than accurate single‐point focusing: it demands reliable wireless power delivery, interference‐free communication, and robust synchronization with real‐time physiological signals [38]. Recent advances in bioelectronic devices have underscored the value of integrated platforms that combine sensing, actuation, wireless interfacing, and physiological feedback [39, 40]. This broader system‐level view is also consistent with emerging studies showing that structured wave–matter interactions can enable hardware‐level functionality across optical and other wave‐based platforms [41, 42].

Our work addresses this challenge by introducing a unified platform that deeply integrates the physics of metasurface‐based ultrasound engineering with the dynamics of real‐time physiological feedback. We propose a new paradigm where the metasurface is not merely a passive lens, but an enabling hardware component within the closed‐loop ultrasonic control architecture. Specifically, this study focuses on three interconnected problems [1]: achieving precise acoustic wavefront manipulation through heterogeneous biological media [2], enabling real‐time synchronized modulation based on physiological feedback, and [3] establishing integrated ultrasonic wireless power transfer and communication without cross‐channel interference. To tackle the first challenge, we developed the Physics‐Constrained Gerchberg‐Saxton (PCGS) method to design the ultrasonic metasurface, which functions as an engineered interface to nonlocally control the ultrasound field. This approach incorporates physical constraints and complex wave interactions directly into the optimization process through a Coupled Angular Spectrum and Finite Element (CASFE) computational framework. This allows for accurate modeling of ultrasound interaction both within the microstructures of the metasurface and through the highly heterogeneous medium, resulting in precise multifocal ultrasound energy delivery according to the desired control functions.

**FIGURE 1:**
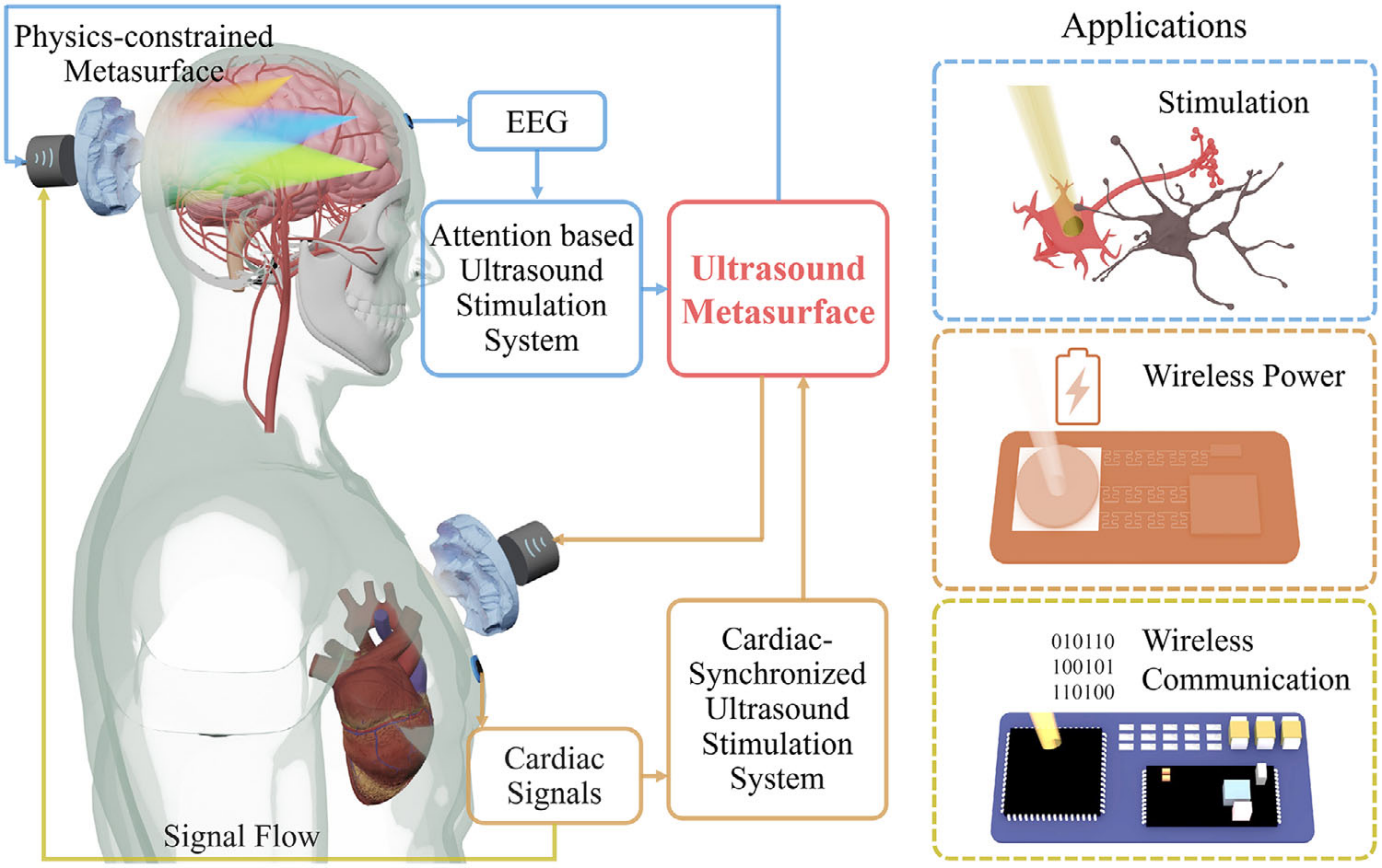
Integrated Ultrasonic Platform for Adaptive Bioelectronic Control with Metasurface. The schematic illustrates the core components of our integrated ultrasound‐based closed‐loop system, encompassing ultrasound wavefront shaping through biological barriers with metasurface, real‐time physiological signal acquisition (EEG/ECG), and multiplexed functionalities including targeted modulation, efficient wireless power delivery, and wireless data communication. This integrated framework enables adaptive, feedback‐driven stimulation for bioelectronic therapies.

Building upon this advanced ultrasound manipulation capability with physics‐constrained metasurface, our research developed an integrated ultrasonic platform for closed‐loop bioelectronic control, as schematically shown in Figure 1. This system incorporates real‐time EEG monitoring and feedback‐driven ultrasound stimulation through a skull phantom. This enables dynamic adjustment of stimulation parameters according to the user's current attentional state. Recognizing analogous acoustic challenges posed by thoracic anatomy, we extended our approach to cardiac applications by designing the metasurface capable of ultrasound manipulation through the ribcage and developing a cardiac‐synchronized ultrasound stimulation system. Finally, to support the development of closed‐loop bioelectronics interfaces, we addressed the critical challenge of integrating ultrasonic wireless power transfer with simultaneous high‐speed data communication. This is achieved by designing a dual‐channel metasurface that supports dual‐frequency operation, with effective co‐focusing at 500 kHz for power transfer and 600 kHz for communication. We validated this integrated platform using real‐time physiological monitoring as a representative application, demonstrating reliable wireless UART transmission of pulse waveforms and calculated metrics despite a substantial 400‐fold power differential between energy transfer and communication signals. Our work bridges a gap between bioelectronic technology, which seeks complex closed‐loop control functions but has been fundamentally limited by rudimentary ultrasound focusing methods, and acoustic metasurfaces, which have developed effective wavefront shaping tools but have yet to be integrated into adaptive, real‐time biological systems. This integrated platform, centered on a physics‐constrained metasurface, establishes a new paradigm where advances in fundamental acoustic physics directly enable next‐generation, intelligent uBMIs and bioelectronic therapies.

## 2 Results

### 2.1 Models and Theory

**FIGURE 2:**
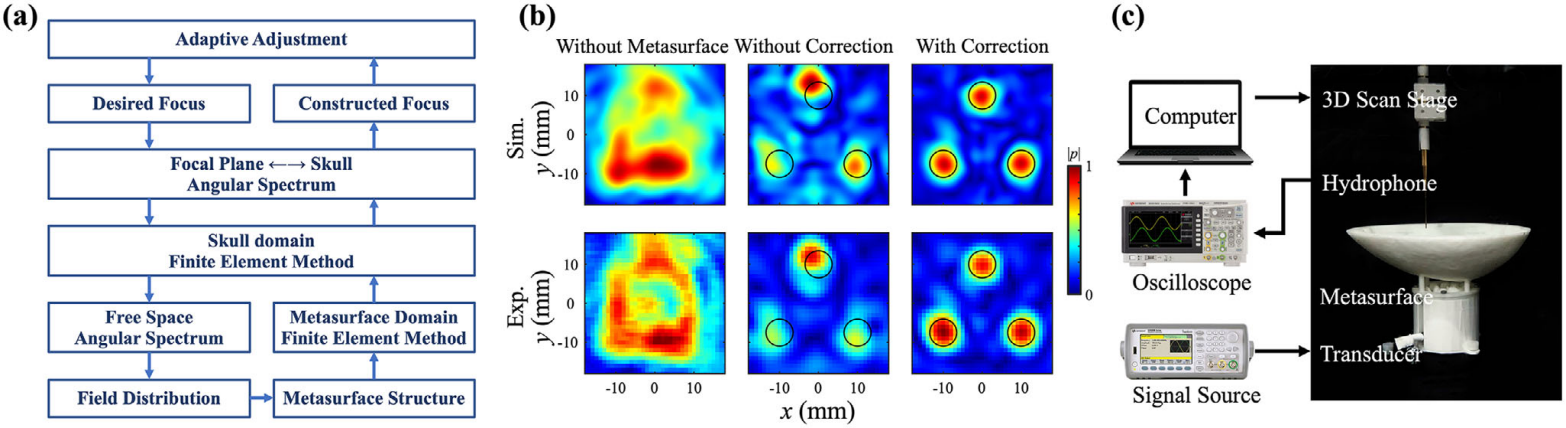
PCGS Algorithm for Transcranial Ultrasound Manipulation. (a) Schematic illustration of the PCGS algorithm workflow, showing the iterative process that incorporates physical constraints into the design of the acoustic metasurface, enabling precise ultrasound wavefront shaping. (b) Experimental validation of the precise wave manipulation through a skull phantom. Ultrasound pressure fields generated by the planar transducer alone (left), by the metasurface without skull correction (middle), and by the metasurface correcting for skull aberrations (right). Black circles indicate the desired focal regions, demonstrating the improvement in focusing achieved with PCGS. (c) Experimental setup for transcranial ultrasound field measurements, showing the configuration used to accurately characterize the ultrasound field after propagation through a human skull phantom.

We designed the metasurface to serve as a core enabling component of the integrated ultrasonic platform for adaptive bioelectronic control. The design process involves two critical challenges, one is converting a plane wave from a planar transducer into a complex, desired ultrasound pattern, and the other is simultaneously correcting the phase aberrations and scattering caused by the biological barrier. The flowchart in Figure 2a illustrates our design process. As a demonstration, we focused on generating a multifocal ultrasound pattern through a skull phantom, whose heterogeneity and irregular geometry significantly degrade the quality and accuracy of ultrasound control. Conventional metasurface design methods, often based on the Gerchberg‐Saxton (GS) algorithm, can partially handle the first task (pattern generation). However, they typically fail to accomplish the second (aberration correction) because they rely on oversimplified phase‐to‐thickness relationships neglecting intricate wave interactions within the metasurface structure or the aberrating biological medium.

To overcome this fundamental limitation, we developed the PCGS algorithm, which departs from the traditional intermediary phase distribution approach, instead shifting to a direct structure‐based design framework. It achieves this by incorporating physical constraints into the iterative optimization process. The PCGS algorithm begins with a backward propagation step, starting from the desired focal pattern on the target plane to derive the geometry of the metasurface on the emission plane. This step leverages our Coupled Angular Spectrum and Finite Element (CASFE) method, which strategically combines the computational efficiency of the angular spectrum method for propagating waves through homogeneous regions, with the physical accuracy of the finite element method (FEM) for modeling wave interactions within complex, heterogeneous structures like the skull. The computational framework employs custom MATLAB scripts [43, 44] and FEM simulations for the forward and backward propagation iterations (detailed implementation provided in Supporting Information). In the forward propagation step, CASFE employs FEM to simulate the acoustic response of the metasurface, rather than relying on a simplified phase‐thickness relationship. A step‐by‐step pseudocode is provided in the Supporting Information. In this framework, PCGS serves as the iterative optimization strategy, whereas CASFE functions as the physical propagation engine used in each forward and backward update. Rather than optimizing an ideal phase mask and converting it afterward, PCGS directly updates a structural thickness map of the metasurface, allowing the optimization to remain tied to a physically realizable device throughout the design process. This full‐wave treatment is critical because it naturally captures nonlocal effects—including diffraction inside the metasurface, lateral coupling between adjacent structural elements, and multiple scattering through the skull. The ultrasound pattern is then adaptively refined using a modified weight‐amplitude method with coefficient *α*: *A*_mod_ = *A*_des_  + α(*A*_des_ − *A*_gen_), where *A*_des_ and *A*_gen_ are the desired and generated amplitudes, respectively. In the present study, a typical value of *α* = 0.5 was used, and the updated amplitude is capped such that *A*_mod_ ≤ 2*A*_des_ to avoid excessive local amplification and promote stable convergence. The PCGS algorithm consistently converges within approximately 20 iterations for multifocal manipulation, with convergence defined as less than 1% variation in focal intensity distribution between successive iterations. On our current workstation, each PCGS iteration requires about 7 min due to repeated MATLAB–COMSOL coupling, while the CASFE strategy substantially reduces the runtime compared with a full‐FEM propagation workflow (details in Supporting Information).

We experimentally validated the precise ultrasound manipulation capability of our metasurface through biological barriers. Our setup involved a 38‐mm‐diameter planar transducer generating a 500 kHz ultrasound wave within a water tank. The designed metasurface was 3D‐printed from photosensitive resin (density: 1.180 g/cm^3^; longitudinal sound speed: 2250 m/s; shear wave speed: 1060 m/s), and closely affixed to the transducer, as illustrated in Figure 2c. A hydrophone (Precision Acoustic, NH0500, 0.5 mm diameter) was employed to measure the 3D acoustic pressure field with a 1 mm step size. To accurately simulate a human skull, we 3D‐printed a skull phantom from the actual CT scan of a human skull, using the same photosensitive resin as the metasurface. All experiments were carried out in a water‐coupled environment to provide a controlled acoustic medium for validating trans‐barrier wavefront correction and integrated system operation. We first measured the acoustic pressure field of the transducer (without the metasurface) to establish the incident boundary condition for our PCGS‐based design. Our target was a three‐focus ultrasound pattern on a transmission plane 50 mm away from the skull outer surface. We then compared the ultrasound pressure fields generated by two metasurface designs: one optimized using our PCGS method with skull correction, and another designed by a conventional GS method without skull correction, as depicted in Figure 2b.

Qualitatively, the PCGS‐designed metasurface maintained notable focus accuracy and intensity uniformity despite the skull's presence. In contrast, the GS‐designed metasurface exhibited obvious field distortions and significant focal shifts. In the simulation results, three‐focal targeting through a skull phantom shows that PCGS correction achieves spatial positioning accuracy of 0.32 ± 0.06 mm compared to 2.27 ± 1.33 mm without correction, representing a 7.1‐fold increase in accuracy. Intensity uniformity across the three foci improved from ±23.8% variation to ±0.6% variation, a 39.8‐fold enhancement. The corrected focal spots also demonstrated improved sharpness with −6 dB focal areas of 48.4 ± 2.98 mm^2^ versus 69.98 ± 25.38 mm^2^ for uncorrected patterns, indicating a 1.45‐fold improvement in spatial confinement. Experimental validation confirms these simulation predictions. PCGS maintains intensity uniformity within ±6.5% across multiple targets through the skull phantom, compared to variations exceeding ±33% without correction. The coefficient of variation was reduced from 28.9% to 6.1%, representing a 78.8% improvement in multifocal intensity uniformity. Spatial accuracy analysis revealed mean positional errors of 0.17 ± 0.29 mm with correction versus 2.38 ± 0.40 mm without correction (see Figure S1 and detailed metrics in Supporting Information). While simulations represent idealized conditions, the experimental results considering fabrication tolerances and material variability agree well with the simulations, which further confirm the robustness of PCGS under practical scenarios. Both datasets confirm substantial improvements in spatial accuracy and intensity uniformity, providing the foundation for applications requiring precise dose distribution across multiple treatment targets. A comprehensive comparison with time‐reversal and phase‐only design strategies is detailed in Table S1 and Figures S2 and S3 of the Supporting Information.

### 2.2 Attention‐Based Closed‐Loop Control of Transcranial Ultrasound Delivery

**FIGURE 3:**
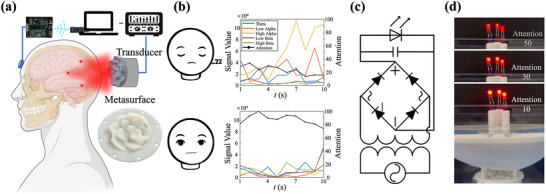
Attention‐based Closed‐Loop Control of Transcranial Ultrasound Delivery. (a) Schematic of the closed‐loop ultrasound stimulation system, showing the TGAM device monitoring brain attention states and the ultrasound transducer delivering targeted stimulation when attention levels drop. The inset shows the fabricated metasurface. (b) Attention state monitoring, showing spectral EEG components (colored lines) and derived attention metrics (black line) during concentrated (top) and distracted (bottom) cognitive states. (c) Circuit diagram of the ultrasonic power system. (d) Experimental demonstration of ultrasound energy transfer through the skull, with LEDs showing varying brightness levels corresponding to different stimulation intensities.

Leveraging the capability of our PCGS‐designed metasurface to generate a precise multifocal ultrasound pattern through the skull, we explored its application in a proof‐of‐concept EEG‐guided transcranial ultrasound delivery scenario. This capability enabled the development of an attention based closed‐loop transcranial ultrasound delivery system that adapts to real‐time brain states, demonstrating an approach that differs from traditional open‐loop methods that apply fixed stimulation regardless of brain activity. Figure 3a illustrates our closed‐loop ultrasound stimulation architecture and includes a photograph of the fabricated passive metasurface. The system continuously monitors EEG signals to assess attentional states, automatically modifying ultrasound parameters when attention metrics fall below predefined threshold values. This adaptive approach enables context‐specific intervention, potentially enhancing the efficacy of targeted stimulation while minimizing unnecessary stimulation during optimal brain states. The PCGS designed metasurface ensures accurate focal patterns despite skull‐induced aberrations, enabling multifocal stimulation, which is a capability typically challenging for conventional single‐focus approaches. Conceptually, multifocal ultrasound delivery may be relevant for future attention‐related neuromodulation scenarios, where cognitive functions like attention allocation, working memory, and executive function rely on the coordinated activation of distributed brain networks, including frontal, parietal, and subcortical regions. By enabling simultaneous and precise targeting of multiple neural nodes, this approach could facilitate modulation of functional connectivity with potentially increased therapeutic efficiency, while possibly reducing stimulation intensity required at individual targets.

For implementation, we used a Think Gear ASIC Module (TGAM) device to acquire frontal EEG signals, as shown in Figure 3a. This module effectively extracts and processes neural oscillations across multiple frequency bands, particularly alpha (8–13 Hz) and beta (13–30 Hz) waves, which are most closely associated with attentional states. Generally, alpha waves reflect relaxed wakefulness and increase as attention wanes, while beta waves rise during focused cognitive tasks, making them key markers of cognitive engagement. The TGAM module integrates these spectral components through proprietary algorithms to generate a normalized attention metric (0‐100 scale), where higher values indicate greater attentional concentration. In this proof‐of‐concept study, we employed a simplified control mechanism where ultrasound stimulation parameters were dynamically adjusted based on real‐time attention metrics. When attention falls below 50, stimulation is initiated at 20% of maximum power, scaling inversely with the attention metric to reach full intensity at an attention level of 0. This design correlates stimulation strength with the degree of detected inattention, providing a framework for tailored intervention. To demonstrate the system's responsiveness, we recorded data during two distinct scenarios: one where a user maintains attention consistently above 65 (concentration) and another where attention remains below 35 (distraction). Figure 3b shows characteristic EEG patterns (spectral power across different bands, represented by colored lines) for concentration (top graphs) and distraction (bottom graphs). The derived attention metric is overlaid (black line). The graphs demonstrate clear differences in both spectral content and the resulting attention metric between concentrated and distracted states, validating the sensitivity of our monitoring approach to distinguish distinct cognitive states and demonstrating the responsiveness of the closed‐loop system.

For experimental safety and visualization, we employed a phantom setup integrated with a wireless ultrasonic power system, as shown in Figure 3c. This circuit converts focused acoustic energy into electrical current via a piezoelectric receiver and incorporates a bridge rectifier for AC‐to‐DC conversion. The harvested energy powers LED indicators, providing visual feedback proportional to the acoustic pressure at the target location. When sustained attention deficits are detected, the system triggers ultrasound stimulation, directing acoustic energy to specific target regions through the skull phantom. Figure 3d demonstrates this LED visualization: when optimal attention levels are detected (above 50), the LEDs remain unilluminated, indicating no intervention. Conversely, when sustained attention deficits are identified, the system automatically triggers ultrasound stimulation, illuminating the LEDs with brightness proportional to the delivered acoustic intensity. This approach not only demonstrates the system's responsiveness to cognitive states but also serves as a quantifiable proxy for acoustic energy delivery through the skull. While this implementation uses LED illumination for mechanism visualization rather than directly demonstrating neural modulation effects, the experiment validates the technical viability of the brain‐controlled ultrasound stimulation paradigm. This closed‐loop demonstration showcases the integration of metasurface‐based ultrasound manipulation into responsive bioelectronic interfaces, establishing the potential for attention‐driven adaptive stimulation. The ability to conditionally modulate neural targets based on ongoing activity is a promising direction for neurological applications, and this work demonstrates that the PCGS‐designed metasurface can overcome acoustic distortions for such a purpose.

### 2.3 Cardiac‐Synchronized Ultrasound Stimulation through Ribcage

To demonstrate the robustness of our physics‐constrained design paradigm across different biological environments, we extended our integrated ultrasonic platform to address the equally challenging and clinically vital domain of the thoracic cavity. The ribcage presents acoustic challenges similar to the skull, fundamentally limiting conventional cardiac ultrasound imaging, which is often restricted to narrow intercostal spaces, thereby limiting the available acoustic window. Similarly, therapeutic ultrasound stimulation faces an analogous constraint as transducer placement is confined to these linear intercostal spaces. This anatomical limitation severely compromises multi‐dimensional targeting capabilities, impeding both spatial precision and comprehensive coverage. For cardiac applications, such as arrhythmia treatment and non‐invasive pacemaking, these challenges are compounded by the need for precise temporal control. Interventions must not only achieve accurate spatial targeting but also deliver stimulation synchronized with specific phases of the cardiac cycle. Therefore, effective ultrasound therapy in the thoracic region critically demands careful correction of acoustic distortions introduced by rib structures to ensure both spatially precise and temporally accurate interventions across cardiac regions.

**FIGURE 4:**
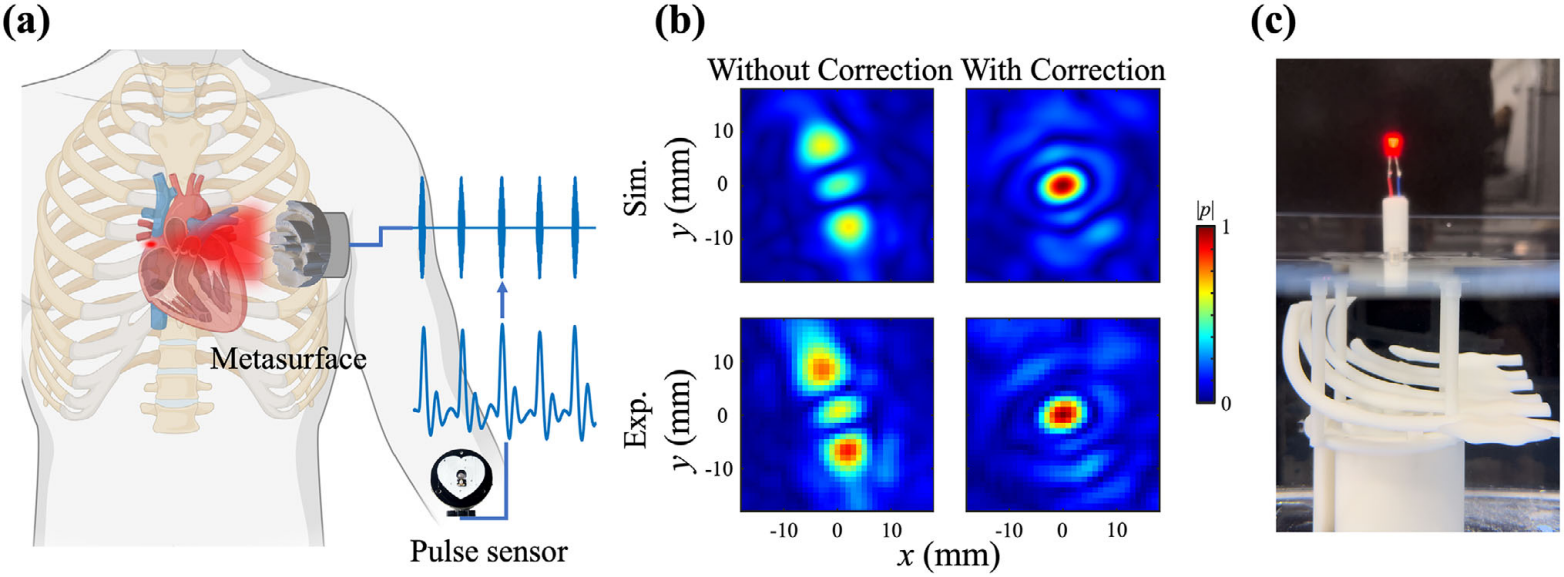
Cardiac‐Synchronized Ultrasound Stimulation Through Ribcage. (a) Schematic representation of the cardiac‐synchronized ultrasound stimulation system. This illustrates how real‐time pulse sensor signals precisely modulate ultrasound emissions, enabling synchronization with specific phases of the cardiac cycle. The inset shows the fabricated metasurface. (b) Experimental validation of ultrasound focusing through a ribcage phantom. The left panels show wavefront distortions and unwanted secondary foci without correction, while the right panels demonstrate the preserved focal pattern achieved with the PCGS‐designed metasurface. (c) Experimental setup showing the cardiac‐synchronized system. An illuminated LED indicator provides visual confirmation of successful synchronization and targeted acoustic energy delivery.

Figure 4a illustrates our approach to cardiac‐synchronized ultrasound stimulation, featuring a photograph of the fabricated passive metasurface that enables enhanced projection through the ribcage. Our approach was designed to address two critical technical requirements for cardiac applications: first, achieving precise focusing through the ribcage's heterogeneous acoustic environment, and second, synchronizing stimulation with cardiac cycles to enhance therapeutic efficacy and safety. While anatomical structures and specific clinical objectives differ between transcranial and cardiac scenarios, the underlying acoustic challenges share similarities. Both the skull and ribcage introduce complex phase aberrations that necessitate iterative wavefront correction methods grounded in similar physical principles. Demonstrating the effectiveness of our PCGS‐based metasurface in these distinct biological contexts underscores its broad adaptability for ultrasound manipulation through complex biological structures. As shown in Figure 4b, the ribcage phantom acts as a periodic acoustic grating, generating multiple diffraction orders due to alternating impedance between ribs and intercostal spaces. Without correction, this grating effect scatters ultrasound energy into undesired secondary foci. Unlike skull‐induced aberrations that primarily displace and attenuate foci, the ribcage redistributes acoustic energy through periodic diffraction. Our PCGS‐designed metasurface compensates these effects by pre‐adjusting the incident wavefront to suppress unwanted diffraction and reinforce the primary focal spot. Quantitatively, experimental measurements confirmed the improvement in focal sharpness, reducing the −6 dB focal area from 108.50 mm^2^ (uncorrected) to 29.00 mm^2^ (PCGS‐corrected), corresponding to a 3.74‐fold reduction in focal area.

Beyond precise spatial targeting, synchronization with the cardiac cycle represents a critical technical feature for developing effective and safe cardiac ultrasound therapies. By incorporating real‐time physiological feedback via a pulse sensor, our system captures cardiac rhythm and dynamically adjusts ultrasound emission parameters via amplitude modulation to align precisely with specific phases of the cardiac cycle. This implementation achieves cycle‐by‐cycle synchronization in the present proof‐of‐concept setup. Figure 4c demonstrates the practical implementation of our system. The ultrasound transducer, driven by pulse‐modulated signals derived from the cardiac feedback loop, delivers precisely timed acoustic energy through a 3D‐printed ribcage phantom to target designated regions. The illumination of an LED acts as a visual confirmation of synchronization success and acoustic energy delivery, validating both temporal precision and acoustic focusing performance within the acoustically challenging thoracic environment. Such temporal control serves two key purposes relevant to cardiac applications. First, it enables the targeting of stimulation to specific phases of the cardiac cycle, which can be critical for applications like arrhythmia treatment, where efficacy and safety may depend heavily on timing relative to the heart's electrical events (e.g., targeting refractory periods or avoiding vulnerable windows). Second, synchronization can effectively compensate for cardiac motion; by consistently triggering stimulation at the same phase in each cycle, the ultrasound focus can maintain a consistent relative anatomical location despite the heart's movement. This principle is analogous to ECG‐gated techniques routinely employed in diagnostic imaging, such as ECG‐gated Cardiovascular Magnetic Resonance Imaging [45] or Cardiac Computed Tomography [46], which precisely mitigate motion artifacts and ensure consistent anatomical assessment across heartbeats. For applications demanding finer control, the system can be configured (e.g., using R‐wave detection from an ECG signal) to introduce specific delays relative to such fiducial markers, enabling precise alignment with desired physiological windows and potentially extending established electrophysiology principles to therapeutic ultrasound.

### 2.4 Integrated Wireless Power Transfer and Data Communication via Ultrasound

**FIGURE 5:**
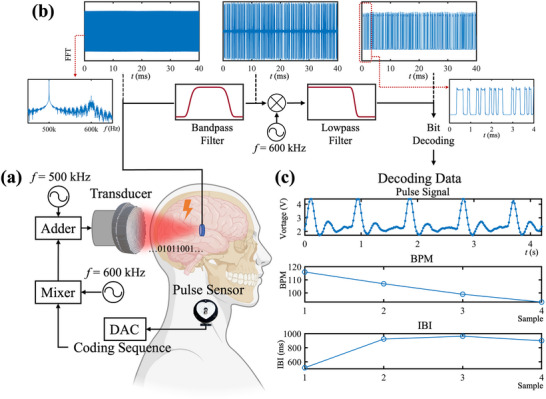
Integrated Ultrasonic Power Transfer and Communication System. (a) System architecture of the dual‐frequency approach, with the 500 kHz channel for continuous wireless power transfer and the 600 kHz channel for data communication, both transmitted simultaneously over a single acoustic path. The inset shows the fabricated metasurface. (b) Signal processing chain displaying representative signal waveforms at various stages of the communication pathway, from raw received signal to filtered and demodulated data, highlighting the effective separation and recovery of the weaker data signal despite a significant power differential. (c) Decoded physiological data transmitted via the ultrasonic communication channel, showing pulse waveform patterns, Inter‐Beat Interval (IBI) measurements, and Beats Per Minute (BPM) trends.

To address the need for reliable wireless power and data transmission in bioelectronic systems, we developed an integrated ultrasonic platform that simultaneously provides wireless power transfer and data communication. Figure 5a illustrates the architecture of our dual‐frequency ultrasonic system, featuring a photograph of the fabricated passive metasurface that enables simultaneous wireless power transfer and communication through a single acoustic pathway for closed‐loop applications. As a practical example representative of data streams in bioelectronic devices, we demonstrate the system's capability using physiological monitoring. Specifically, a pulse sensor captures the subject's pulse waveform and generates an analog voltage signal proportional to blood flow. This analog signal is then digitally processed by an Arduino UNO running a peak detection algorithm. The algorithm calculates the Inter‐Beat Interval (IBI) by measuring time differences between consecutive pulse peaks and derives the Beats Per Minute (BPM) by averaging the ten most recent intervals. The processed data—including raw pulse waveforms sampled at 50 Hz, calculated IBI, and BPM values—are transmitted through the Arduino's UART interface at a baud rate of 9600. The digital data stream from the microcontroller is fed into a mixer circuit that modulates the information onto a 600 kHz ultrasonic carrier using Amplitude Shift Keying [17, 47]. Simultaneously, a separate 500 kHz continuous wave is generated for power transfer. These two operating frequencies were selected to remain within the effective bandwidth of the transducer while providing sufficient spectral separation for filtering and crosstalk suppression. On the receiver side, we implemented parallel processing paths optimized for their respective functions. The power harvesting path directly connects the receiving transducer output to a full‐wave bridge rectifier, efficiently converting the 500 kHz acoustic energy into stable DC power. The communication path is tapped from the same transducer output through a high‐impedance connection, which minimizes electrical loading and preserves efficiency of power harvesting.

In our experiments, we drove the transmitting transducer with a 20 V signal at 500 kHz for power transfer and a 1 V signal at 600 kHz for data communication. This setup results in a transmitted power ratio of approximately 400:1 between the power and data carriers, demonstrating the system's wide dynamic range. As depicted in Figure 5b, the raw received signal in the time domain predominantly features the high‐power component intended for energy transfer. However, spectral analysis reveals the much weaker data component with discernible spectrum spreading around 600 kHz. This significant power differential is highly relevant for implantable devices, where energy harvesting demands substantially higher power levels, while data communication can reliably operate with much lower signal strength. Despite this large power disparity, our system recovered physiological data without significant interference from the dominant power signal, as demonstrated by consistent waveform and metric recovery. The signal processing chain begins with a sixth‐order bandpass filter centered at 600 kHz with a 60 kHz bandwidth. This filter provides over 33 dB of attenuation at 500 kHz, thereby effectively isolating the data carrier from the stronger power signal. Following filtration, the signal undergoes quadrature demodulation using in‐phase and quadrature reference signals at 600 kHz. The demodulated signal then passes through a fourth‐order low‐pass filter (cutoff frequency set to six times the baud rate) to remove high‐frequency artifacts while preserving the digital information content. The final stage applies standard UART protocol decoding to recover the original physiological data, including pulse waveforms, IBI, and BPM values as shown in Figure 5c. The present communication experiment is intended as a proof‐of‐concept demonstration rather than a full communication‐link benchmark. Future studies should further evaluate BER or packet‐error rate under varying channel conditions. Additional transcranial field analyses at both 500 and 600 kHz in Figure S4 show that, without the metasurface, the transmitted acoustic energy remains diffuse after skull propagation, whereas the metasurface concentrates both frequencies at the target position. Consistently, wireless power transfer measurements in Figure S5 show that the metasurface produces substantially higher rectified DC voltages than the uncorrected case across the tested driving range, including a 6.9‐fold increase at 20 Vpp (6.016 V vs 0.869 V).

Compared to conventional single‐frequency time‐multiplexed approaches, our dual‐frequency method enables continuous energy delivery without interrupting data transmission. This is a clear advantage for implantable devices requiring consistent power supply alongside continuous communication capability. Furthermore, this approach requires only a single pair of ultrasonic transducers for both power and data functions, reducing the overall footprint compared to dual‐transducer systems or separate inductive/RF coils. This architectural efficiency is particularly valuable for size‐constrained implantable applications where component miniaturization is essential.

## 3 Conclusions

In this study, we have presented a holistic framework that leverages the unique properties of ultrasound to advance closed‐loop systems wireless bioelectronics and emerging ultrasonic brain‐machine interfaces. Our core contributions span three interconnected and foundational areas [1]: developing the Physics‐Constrained Gerchberg‐Saxton methodology for high‐precision and flexible ultrasound manipulation through complex biological media [2], demonstrating closed‐loop systems for both transcranial and cardiac‐synchronized ultrasound delivery that integrate real‐time physiological feedback to enable adaptive, context‐aware stimulation, and [3] developing an integrated ultrasonic platform for simultaneous wireless power transfer and data communication via a single acoustic pathway.

The PCGS algorithm marks an advance in transcranial and transthoracic ultrasound manipulation. It directly incorporates comprehensive physical constraints and actual material properties into the optimization process. Unlike traditional methods relying on oversimplified phase‐to‐thickness relationships, our approach models complex wave interactions within both the metasurface structure and heterogeneous biological barriers. This results in multifocal patterns with consistent intensity distribution in experiments (variations within ±6.5% compared to over ±30% in uncorrected approaches) and precise spatial targeting even through acoustically complex structures like the skull and ribcage. Our physiologically‐synchronized closed‐loop systems demonstrate the practical application of this manipulation capability. The attention‐based ultrasound stimulation system continuously monitors EEG signals to modulate ultrasound parameters in response to cognitive states. Similarly, the cardiac‐synchronized system aligns ultrasound energy delivery with specific phases of the cardiac cycle. These implementations illustrate how real‐time physiological feedback can enhance both the precision and potential efficacy of ultrasonic interventions by delivering stimulation only when and where it is most beneficial. To support sustainable operation vital for these closed‐loop systems, our dual‐frequency ultrasonic platform (500 kHz for power, 600 kHz for data) enables simultaneous wireless power transfer and wireless data communication through a single acoustic path. This approach maintains data integrity despite a 400:1 power differential between channels, eliminating the need for time‐multiplexing and reducing the system complexity compared to dual‐transducer systems or separate electromagnetic components. It should be noted that the metasurface in the current system is passive and static; the demonstrated closed‐loop adaptivity is realized through real‐time modulation of stimulation amplitude and/or temporal gating, while the spatial focal pattern remains fixed by the metasurface design.

While our results demonstrate promising capabilities, several critical steps remain on the path to widespread clinical translation. These include rigorously addressing inter‐individual anatomical variability, accounting for dynamic tissue properties, and further miniaturizing components for implantable applications. In addition, supplementary simulations in Figure S6 using representative bone‐like skull parameters show that the PCGS/CASFE framework can work under realistic skull‐like conditions and retains reasonable robustness to moderate sound‐speed mismatch. Future work will focus on comprehensive in vivo validation across different biological contexts, including the rigorous assessment of thermal effects, cavitation thresholds, and compliance with regulatory safety guidelines, as well as optimization for specific therapeutic applications. By addressing the core challenges of ultrasound control accuracy, feedback integration, and wireless functionality, our work lays the foundation for translating ultrasound technologies into impactful clinical and research tools, with potential applications in cognitive enhancement, neurological disorder treatment, cardiac therapy, and the development of autonomous bioelectronic systems. Additionally, integrating active or reconfigurable elements into the metasurface could enable dynamic, real‐time ultrasound adjustment. This would allow the system to adapt not only stimulation intensity and timing, but also spatial focal targeting in response to evolving physiological states or changing anatomical conditions. Such programmable acoustic metasurfaces could ultimately lead to intelligent, spatiotemporally adaptive bioelectronic interfaces, akin to concepts explored in electromagnetic smart metasurfaces and reconfigurable intelligent surfaces [48, 49].

## Conflicts of Interest

The authors declare no conflicts of interest.

## Supporting information




**Supporting File**: advs75563‐sup‐0001‐SuppMat.docx.

## Data Availability

The data that support the findings of this study are available from the corresponding author upon reasonable request.
