# Supplementary material for: Integrated Ultrasonic Platform for Bioelectronic Control through Biological Barriers Based on Metasurface
Source: Adv Sci (Weinh). 2026 May 7;13(43):e75563. doi: 10.1002/advs.75563 (PMC13335894; doi:10.1002/advs.75563)
Supplement: Supplementary file 1 — Supporting File: advs75563‐sup‐0001‐SuppMat.docx. [file ADVS-13-e75563-s001.docx]

**Supplementary**

**Integrated Ultrasonic Platform for Bioelectronic Control Through Biological Barriers Based on Metasurface**

Chuanxin Zhang^1^, Hanjie Xiao^1^, Xue Jiang^1,2,*^, and Dean Ta^1,2,*^

^1^College of Biomedical Engineering, Fudan University, Shanghai, 200433, China.

^2^State Key Laboratory of Integrated Chips and System, Fudan University, Shanghai, 200433, China.

*Corresponding author. Email: xuejiang@fudan.edu.cn (X.J.); tda@fudan.edu.cn (DA.T.)

**Detailed Implementation of the PCGS Algorithm**

The Physics-Constrained Gerchberg-Saxton (PCGS) algorithm provides a robust framework for designing acoustic metasurfaces capable of precise wavefront shaping through complex biological media. The iterative process is underpinned by our Coupled Angular Spectrum and Finite Element (CASFE) computational method.

In the present implementation, the metasurface is not represented as a free-form voxelized 3D topology, but as a fabrication-oriented discretized thickness map defined on a 2D lateral grid. The active metasurface aperture is circular with a radius of 19 mm, embedded in a square computational design window of width 44 mm. The final geometric update is performed on a 1 mm lateral sampling grid, which is consistent with our fabrication-compatible parameterization. On this grid, the number of independently updated geometric elements is approximately the number of sampled pixels inside the circular aperture, i.e., *N* ≈ *π* × 19^2^ ≈ 1134. So in practice the design contains on the order of 10^3^ independent thickness pixels. Each pixel is assigned one geometric parameter, namely its local thickness *t*(*x*,*y*). After backward propagation, the complex field at the metasurface plane is used to derive an updated fabrication-compatible thickness map, *t*$\left( x,y \right)=\Delta\varphi\left( x,y \right)/\left( k_{w}-k_{m} \right)$, where $k_{m}$and $k_{w}$​ are the wavenumbers in the metasurface and water respectively. In practice, this update uses the local phase requirement as an intermediate guide for structural revision, while the actual forward evaluation of the metasurface response is always performed through full-wave simulation rather than an ideal phase-only model. The thickness is constrained between 0.25 mm and 8.75 mm, corresponding to 35 discrete thickness states with a step size of 0.25 mm. In this way, the optimization remains directly tied to a physically realizable metasurface geometry throughout the iterative loop, rather than optimizing an ideal phase profile first and realizing it only afterward.

The algorithm begins with a backward propagation step to derive an initial metasurface geometry from the desired focal pattern. This process relies on the CASFE method to accurately model wave propagation through the heterogeneous medium. Specifically, the angular spectrum (AS) method is first employed to calculate the acoustic field propagation from the target focal plane back to the boundary of the skull phantom. The resulting complex pressure field on this surface then serves as the input boundary condition for a subsequent finite element method (FEM) simulation. The use of FEM is critical as it accommodates the irregular mesh structures of the skull’s complex geometry and accurately captures physical phenomena such as acoustic-elastic coupling at material interfaces, which are challenging for methods restricted to regular grids. Acoustic attenuation is incorporated implicitly through the full-wave propagation engine rather than using a separate intensity-restoration term. After propagating through the skull via FEM, the acoustic field is then propagated the remaining distance to the source plane using the AS method.

In the forward propagation phase, the CASFE method is again employed. Here, FEM simulates the acoustic response of the metasurface structure itself, replacing idealized thickness-to-phase approximations. Unlike waveguide approximations that treat each unit cell independently, the FEM-based metasurface calculation solves the full acoustic wave equation over the entire structured domain. As a result, nonlocal effects—including diffraction within the metasurface, lateral coupling between adjacent thickness elements, and multiple scattering events inside the structure—are naturally captured. This step ensures that the model accounts for intricate wave interactions within the metasurface's microstructures. Following forward propagation through the metasurface and the skull phantom, the resulting focal pattern is compared to the desired pattern.

To accelerate convergence and improve focusing quality, the generated pattern is refined using a modified weight-amplitude method. This introduces a coefficient α to adjust the amplitude at each focus as: $A_{\mathrm{mod}}=A_{\mathrm{des}}+\alpha(A_{\mathrm{des}}-A_{\mathrm{gen}})$, where $A_{\mathrm{des}}$ and $A_{\mathrm{gen}}$ are the desired and generated amplitudes, respectively, thereby suppressing over dominant peaks and compensating underpowered foci to enhance uniformity. In the present implementation, a typical value of *α*=0.5 is used, and the updated amplitude is capped such that $A_{\mathrm{mod}}\boldsymbol{\leq}2A_{\mathrm{des}}$ to avoid excessive local amplification and promote stable convergence. This value was selected empirically because it provided stable convergence and good multifocal uniformity across the representative design cases studied here.This adaptation helps to better balance intensity across multiple foci and improve convergence compared to standard amplitude-replacement used in the conventional GS algorithm. Focal positions are also algorithmically adjusted based on observed deviations from the desired target locations. The target focal coordinates are predefined prior to optimization and remain fixed throughout the iterative process. The algorithm does not update the prescribed target positions; instead, it updates the amplitude weights within the predefined finite-area target masks. Because each target is represented by a spatial mask rather than an ideal single-pixel point, a nonuniform amplitude distribution within the mask may produce an apparent shift in the centroid of the generated focal region. This effect reflects imperfect field matching inside the target mask rather than an actual change of the prescribed focal coordinates.

The entire computational framework employs **custom MATLAB scripts** for the AS calculations, which are integrated with COMSOL Multiphysics for the FEM simulations. To ensure precise wave propagation modeling, a maximum mesh element size of λ/6 (where λ is the acoustic wavelength) is maintained. Data exchange between these two computational domains is facilitated through the Live Link for COMSOL with MATLAB, which passes the complex acoustic pressure field across the simulation interfaces to maintain field continuity. This iterative process of physics-informed propagation and refinement optimizes the metasurface design by improving the correspondence between the simulated design and the physically realizable acoustic field at the focal plane. The algorithm's convergence was defined by less than a 1% change in the focal intensity distribution between successive iterations, a criterion that was typically met within approximately 20 iterations for multifocal applications. More specifically, convergence was evaluated using the normalized intensity distribution across all predefined target masks.The relatively rapid convergence, despite the high-dimensional design space, can be attributed to three factors. First, the optimization is strongly physics-guided: the target field is back-propagated through the actual propagation model, so each iteration starts from a physically meaningful estimate rather than a random search point. Second, the structural parameterization is deliberately constrained and fabrication-oriented, which makes the effective search space far more structured than in unconstrained free-form topology optimization. Third, the modified amplitude update rule actively suppresses over-dominant focal peaks and boosts underpowered ones, thereby preventing the iteration from stagnating around imbalanced multifocal solutions.

In the backward step, the updated complex target field at the focal plane is first propagated in MATLAB to the skull-side interface using the angular spectrum method. The resulting complex pressure distribution is then exported from MATLAB and imported into COMSOL as interpolation functions representing the spatially varying complex boundary condition. COMSOL subsequently solves the backward wave propagation through the skull domain. The computed complex pressure field on the source-side boundary is then exported back to MATLAB, where the remaining propagation to the metasurface plane is completed by AS calculation. At this stage, MATLAB performs the structural update by extracting the required phase response, converting it into a fabrication-compatible thickness map, and applying the prescribed quantization and geometric constraints. In the forward step, the updated thickness map is passed back to COMSOL, where it is used to reconstruct the metasurface geometry parametrically, followed by automatic remeshing of the structured domain. COMSOL then performs the full-wave forward simulation through the metasurface and the skull, thereby accounting for diffraction inside the metasurface, lateral coupling between neighboring structural elements, and scattering at the heterogeneous barrier. The resulting complex pressure field is exported again to MATLAB, which completes the remaining propagation to the target plane and evaluates the generated focal pattern for the next iteration. To ensure continuity between the two computational environments, the complex acoustic field is transferred in terms of its spatially sampled amplitude and phase, using interpolation functions generated from exported matrices. This allows the MATLAB-based AS solver and the COMSOL-based FEM solver to operate as complementary modules within a single iterative loop.

The dominant computational cost arises from the repeated MATLAB–COMSOL coupling, particularly the FEM solves in the backward and forward propagation steps. The AS calculations in MATLAB typically require only seconds, whereas the FEM simulations and inter-software data exchange dominate the overall runtime. On a workstation equipped with an AMD 5900X CPU and 128 GB RAM, a representative multifocal design required approximately 170 s for the backward propagation step and 246 s for the forward propagation step in each PCGS iteration, corresponding to about 416 s per iteration. For a typical design converging in around 20 iterations, the total runtime was therefore approximately 138 min. For comparison, if the same iterative procedure is implemented using FEM over the full propagation path without the CASFE acceleration strategy, the backward and forward steps each require about 480 s, corresponding to about 960 s per iteration and about 320 min for 20 iterations. This comparison highlights the computational advantage of CASFE, which reduces the runtime by more than half while preserving the physical fidelity required for trans-barrier acoustic design. The exact runtime may still vary depending on mesh density, solver settings, and barrier geometry complexity.

All experimental validations in this work were carried out in a water-coupled environment, which provided a controlled acoustic medium for evaluating the metasurface design and integrated system performance.

**PCGS Algorithm**

**Input:**

- Desired focal-region mask **A_mask**, target amplitude **A_des**
- Update coefficient α = 0.5, convergence threshold ε = 1%
- Maximum iterations N_iter

**Output:** Metasurface thickness map **T**

**Initialization:**

 Initialize target complex field: **P**₁₁ ← **A_des** · e^(j·0)

 Initialize amplitude weight: **W** ← **A_des**

*(Design grid: 1 mm; Propagation grid: 0.25 mm)*

**for** i = 1 **to** N_iter **do**

*// ——— Backward Pass (Target → Metasurface) ———*

**P**₀₁ ← ASM_Backward(**P**₁₁, z₁)

*(Angular Spectrum: target plane → skull boundary)*

 Export ∠**P**₀₁, |**P**₀₁| → COMSOL interpolation functions

**P**₁₀ ← FEM_Backward(M_skull, **P**₀₁)

*(COMSOL: backward FEM through skull)*

 Import **P**₁₀ ← COMSOL pressure export

**P**₀₀ ← ASM_Backward(**P**₁₀, z₀)

*(Angular Spectrum: skull boundary → metasurface plane)*

*// ——— Phase-to-Structure Mapping ———*

 Extract phase: **φ** ← ∠**P**₀₀

 Downsample **φ** to design grid (1 mm) via bilinear interpolation

 Compute thickness: **T**_raw ← **φ** / (k_w − k_m)

 Quantize: **T** ← Quantize(**T**_raw, 0.25 mm, 8.75 mm, Δt = 0.25 mm)

*(Discrete fabrication-compatible levels)*

*// ——— Forward Pass (Metasurface → Target) ———*

 Export **T** → COMSOL interpolation function

**P**₀₁^fwd ← FEM_Forward(**T**, M_skull)

*(COMSOL: forward FEM through metasurface + skull)*

 Import **P**₀₁^fwd ← COMSOL pressure export

**P**₁₁^gen ← ASM_Forward(**P**₀₁^fwd, z₁)

*(Angular Spectrum: skull boundary → target plane)*

*// ——— Adaptive Amplitude Reweighting ———*

**A**_gen ← |**P**₁₁^gen| · **A_mask**

*(Extract generated amplitude within focal-region masks)*

**A**_mod ← **A**_des + α · (**A**_des − **A**_gen)   *(α = 0.5)*

 Apply cap: **A**_mod ← min(**A**_mod, 2 · **A**_des)   
 *(Prevent overcompensation)*

 Reconstruct target field:  **P**₁₁ ← **A**_mod · e^(j · ∠**P**₁₁^gen)

*// ——— Convergence Check ———*

**I**_gen ← Normalize( |**P₁₁**^gen|^2 within all predefined target masks )

**if** i > 1 **and** max( |**I**_gen^(i) − **I**_gen^(i−1)| ) < ε then

**break**

**end if**

**end for**

**return T**

**MATLAB–COMSOL Data Exchange**

MATLAB COMSOL

│ │

│ │

│ writematrix(phase / amplitude) │

│ ────────────────────────────────────► │ Interpolation functions

│ │

│ │ FEM solve: backward propagation

│ │ through skull

│ │

│ readmatrix(backward field) │ Export pressure field

│ ◄──────────────────────────────────── │ (backward field P₁₀)

│ │

│ │

│ writematrix(thickness map) │

│ ────────────────────────────────────► │ Interpolation function

│ │ (thickness T)

│ │

│ │ FEM solve: forward propagation

│ │ through metasurface + skull

│ │

│ readmatrix(forward field) │ Export pressure field

│ ◄──────────────────────────────────── │ (forward field P₀₁^fwd)

│ │

**Experimental Transcranial Multifocal Ultrasound Performance**

Figure S1(a) presents experimental cross-sectional intensity profiles along the focal plane, revealing the dramatic improvement achieved by PCGS correction. The corrected beam patterns (red solid lines) demonstrate well-defined, symmetric focal peaks with minimal sidelobe interference, closely matching the intended multifocal configuration. In stark contrast, uncorrected patterns (blue dashed lines) exhibit severely distorted focal shapes, significant peak displacement, and substantial intensity variations between target locations.

Quantitative analysis of the three focal intensities, normalized to the maximum value, yielded values of 0.885, 1.000, and 0.955 with PCGS correction, corresponding to a mean intensity of 0.947 ± 0.058 and a coefficient of variation of 6.12%. The maximum deviation from the mean intensity was ±6.52%. These results confirm high uniformity across the foci. Without correction, normalized focal intensities measured 0.915, 0.549, and 0.599 (mean: 0.688 ± 0.199), yielding a coefficient of variation of 28.87% and maximum deviation of ±33.08%. The PCGS implementation thus produced a 78.8% reduction in intensity variability and an 80.3% improvement in maximum deviation.

The effectiveness of the PCGS correction is particularly evident in the dramatic improvement of the weakest focal spots. While the uncorrected system produced two severely attenuated foci with intensities of only 54.9% and 59.9% relative to the strongest focus, PCGS correction maintained all three foci above 88.5% of the maximum intensity. This consistent intensity delivery across multiple targets is essential for clinical applications requiring uniform therapeutic dose distribution.

Spatial accuracy analysis revealed positional errors of 0.17 ± 0.29 mm with correction versus 2.38 ± 0.40 mm without correction, demonstrating a 92.9% improvement in targeting precision. The enhanced spatial and intensity control achieved by the PCGS correction is critical for clinical applications requiring precise dose distribution across multiple treatment targets.

**Transcostal Single-focal Ultrasound Characteristics**

Figure S1(b) illustrates the acoustic field behavior for transcostal ultrasound focusing, where the ribcage presents a fundamentally different aberration mechanism compared to skull transmission. The ribcage acts as a periodic acoustic grating structure, creating multiple diffraction orders that manifest as characteristic interference patterns in the axial (*y*-direction) beam profiles.

The corrected transcostal pattern (red solid line) shows a well-focused central peak with controlled sidelobe levels, indicating effective compensation for rib-induced phase aberrations. However, the uncorrected pattern (blue dashed line) exhibits the classic signatures of grating diffraction: multiple subsidiary peaks at regular spatial intervals corresponding to higher-order diffraction modes, and significant energy redistribution away from the intended focal region.

Unlike the skull case where aberrations primarily cause focal displacement and broadening with severe intensity attenuation (as evidenced by the 35-50% intensity loss in the weakest foci), the ribcage introduces periodic phase modulation that generates a multi-order diffraction pattern. This grating-like behavior results from the alternating acoustic impedance between ribs and intercostal spaces, creating a spatially periodic transmission function. The PCGS correction algorithm successfully mitigates these diffraction artifacts by pre-compensating the incident wavefront to destructively interfere with unwanted diffraction orders while constructively reinforcing the primary focal spot. Quantitatively, PCGS correction reduced the -6 dB focal area from 108.50 mm² (uncorrected) to 29.00 mm² (corrected), representing a 3.74-fold enhancement in spatial confinement.

The effectiveness of the PCGS algorithm in correcting for these two distinct types of aberrations, complex, irregular phase distortions from the skull and periodic diffraction from the ribcage, which highlights its versatility for addressing diverse anatomical challenges in therapeutic ultrasound.


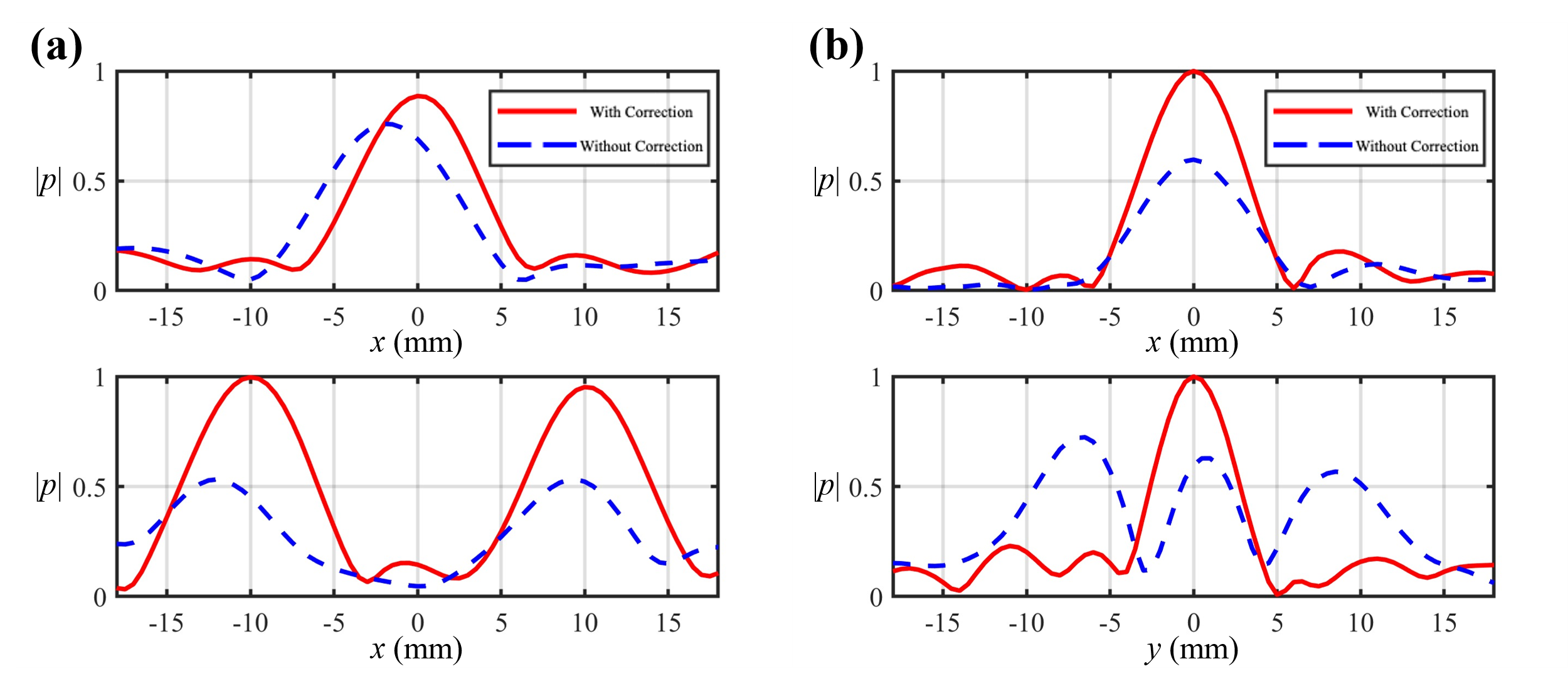


**Figure S1.** **Experimental ultrasound focusing performance with and without PCGS correction. (a)** Transcranial three-focal cross-sectional intensity profiles showing PCGS correction (red) vs. uncorrected patterns (blue). **(b)** Transcostal single-focal cross-sectional intensity profiles demonstrating grating diffraction mitigation. Scale bars indicate spatial dimensions; intensities normalized to maximum.

**Comparison With Conventional Transcranial Design Strategies**

To further clarify the necessity of the physics-constrained design, we compared our method with two commonly used strategies in transcranial acoustic design: time reversal and a conventional iterative phase retrieval algorithm. In the time-reversal approach, a virtual source is placed at each target position, and the backward-propagated wave is recorded at the metasurface plane; the extracted phase is then used for structural design. Although time reversal improves spatial localization, it does not explicitly balance the relative amplitudes among multiple prescribed foci, leading to poor multifocal uniformity. In the phase-only approach, the skull is included in the propagation path, and a conventional iterative phase retrieval algorithm is used to optimize the ideal phase modulation.

As shown in Fig. S2(b), the phase-only iterative method can produce relatively uniform multifocal intensity when evaluated under ideal phase modulation. However, once the designed phase is converted into a realizable metasurface thickness distribution through the phase-to-structure mapping and the resulting structure is re-evaluated using full-wave simulation, the focal uniformity is reduced, as shown in Fig. S2(c). This discrepancy indicates that phase-level optimization alone does not fully capture the non-local wave effects introduced by structural realization, including diffraction and coupling within the metasurface. This comparison supports the motivation for introducing the physics-constrained design strategy.

To provide a clearer quantitative comparison, Table S1 summarizes the performance of different transcranial multifocal design strategies. Compared with the time-reversal approach, PCGS maintains comparable spatial localization while substantially improving multifocal intensity balance. Specifically, the spatial positioning accuracy is improved from 0.44 ± 0.16 mm to 0.32 ± 0.06 mm, while the intensity variation across the three foci is reduced from ±30.2% to ±0.6%. The -6 dB focal area is also reduced from 52.52 ± 8.57 mm² to 48.40 ± 2.98 mm². In addition, although the conventional phase-only optimization yields excellent performance under ideal phase modulation, its performance degrades markedly after conversion to a realizable structure, with the spatial positioning error increasing from 0.08 ± 0.14 mm to 1.39 ± 0.36 mm, the intensity variation increasing from ±0.2% to ±12.9%, and the -6 dB focal area expanding from 47.40 ± 7.54 mm² to 55.27 ± 8.57 mm². These results further support the motivation for introducing the physics-constrained design strategy, which directly incorporates structural realizability and non-local wave interactions into the optimization loop.


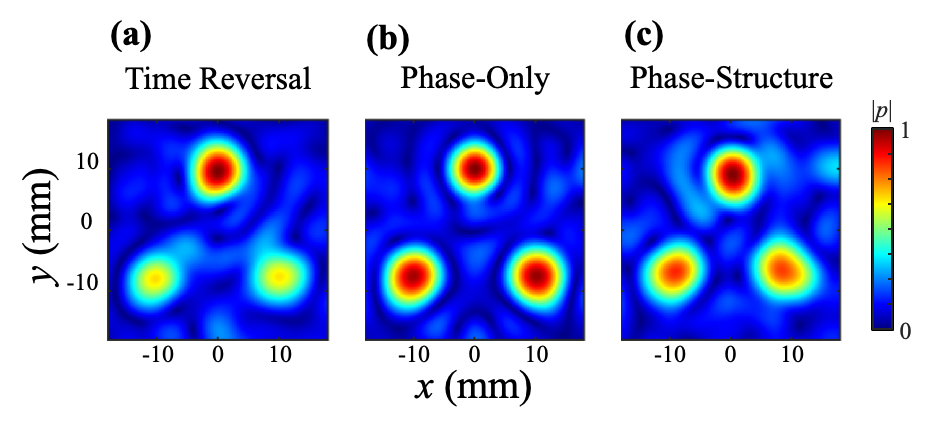


**Figure S2.** **Comparison of different design strategies for transcranial multifocal focusing. (a)** Time reversal result for generating the target multifocal pattern through the skull. **(b)** Phase-only iterative result under ideal phase modulation, showing uniform focal intensities before structural realization. **(c)** Full-wave result after phase-to-structure conversion of the phase-only design, showing degraded focal uniformity after structural realization.


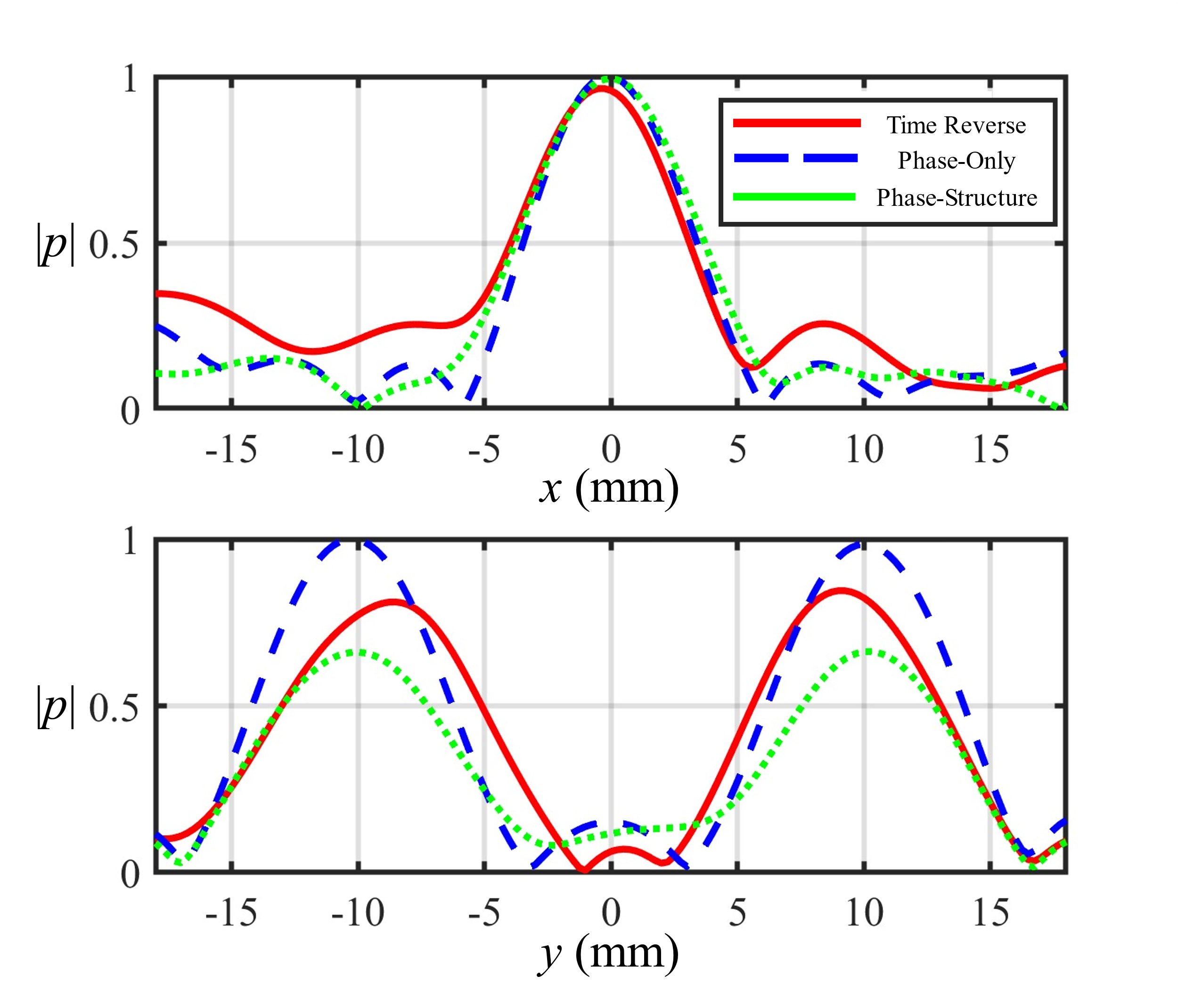


**Figure S3.** **Ultrasound focusing performance across different design strategies.**

**Table S1. Quantitative comparison of simulated transcranial multifocal design strategies**

| **Design strategy** | **Spatial positioning accuracy (mm)** | **Intensity uniformity** | **-6 dB focal area (mm²)** | **Description** |
| --- | --- | --- | --- | --- |
| Uncorrected | 2.27 ± 1.33 | ±23.8% | 69.98 ± 25.38 mm² | No skull-aberration correction |
| Time-reversal correction | 0.44 ± 0.16 | ±30.2% | 52.52 ± 5.06 mm² | Back-propagation-based correction without the full physics-constrained structural optimization |
| Phase-only design | 0.08 ± 0.14 | ±0.2% | 47.40 ± 7.54 mm² | Ideal phase-only modulation before structural realization |
| Phase-structure realization | 1.39 ± 0.36 | ±12.9% | 55.27 ± 8.57 mm² | Full-wave re-evaluation after converting the ideal phase profile into a metasurface structure |
| **PCGS**  **(this work)** | **0.32 ± 0.06** | **±0.6%** | **48.40 ± 2.98 mm²** | Direct physics-constrained structural optimization with CASFE |

**Acoustic and Electrical Validation of the Dual-Frequency Ultrasonic Link**

To provide a more direct validation of the dual-frequency ultrasonic link, we further examined both the transcranial acoustic field distributions and the wireless power transfer performance of the system.

As shown in Fig. S4, the acoustic field distributions after propagation through the skull were evaluated at the two operating frequencies, with the acoustic pressure amplitude normalized to the maximum value at each frequency. Without the metasurface, where the transducer directly irradiates through the skull, the acoustic fields at both 500 kHz and 600 kHz are diffuse, and the pressure amplitude at the target region is relatively low, which is unfavorable for reliable power delivery and communication. In contrast, when the transcranial metasurface is introduced, both frequencies are focused at the target position (0,0). This enables accurate alignment with the implanted device, while improving energy localization and reducing unintended exposure to surrounding tissues.


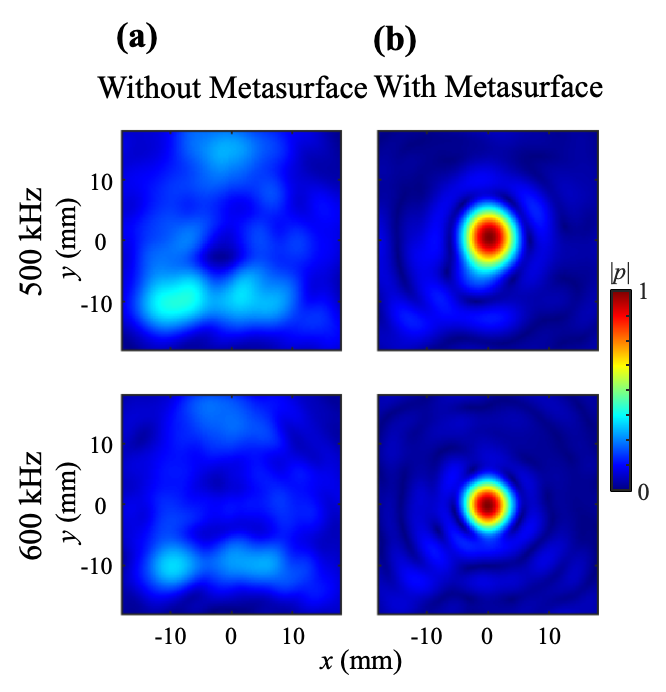


**Figure S4.** **Simulated transcranial acoustic fields of the dual-frequency system with and without the metasurface. (a)** Normalized acoustic field distributions at 500 kHz and 600 kHz without the metasurface, showing diffuse energy after propagation through the skull. **(b)** Normalized acoustic field distributions at 500 kHz and 600 kHz with the metasurface, showing focused energy at the target position.

To quantify the contribution of the metasurface to wireless power transfer, we further compared the rectified voltage at the receiver with and without the metasurface. As shown in Fig. S5(a), under a driving voltage of 20 Vpp, the metasurface system produces a stable rectified output of approximately 6.016 V, whereas the system without the metasurface yields only 0.869 V. Fig. S5(b) further summarizes the received voltage over a driving range of 5–30 Vpp. The metasurface-assisted case outperforms the metasurface-free case at all tested voltages, indicating that the transcranial metasurface markedly improves acoustic power delivery efficiency. This enhancement reduces the transmitter voltage required to reach a given operating voltage at the receiver and is therefore important for practical implantable bioelectronic applications.


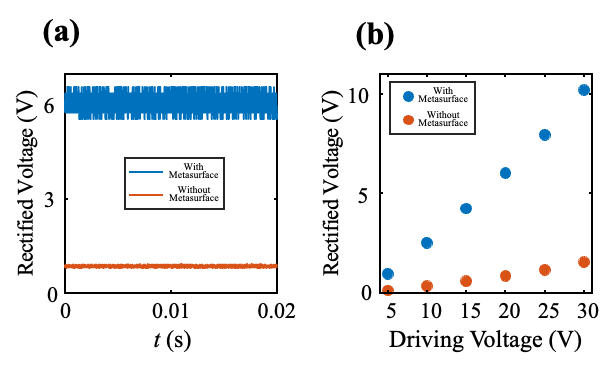


**Figure S5.** **Experimental comparison of wireless power transfer performance with and without the metasurface. (a)** Rectified output voltage waveforms under 20 Vpp excitation with and without the metasurface. **(b)** Average rectified output voltage as a function of driving voltage from 5 to 30 Vpp, showing higher received voltage with the metasurface across the full range.

**Sensitivity to Skull Acoustic Properties and Robustness to Model Mismatch**

To further examine the translational relevance of the proposed framework, we performed an additional simulation study using representative bone-like acoustic parameters for the skull model. In the main experiments, the skull phantom was fabricated from photosensitive resin to provide a controlled proof-of-concept validation environment. To evaluate whether the PCGS/CASFE workflow can be extended beyond this specific phantom condition, we reassigned the skull model with representative bone-like parameters (density: 1.850 g/cm³ and sound speed: 2700 m/s) and re-designed the metasurface using the same optimization framework.

As shown in Figure S6, the metasurface remains capable of generating the prescribed three-focus pattern under this more challenging propagation condition. This result indicates that the proposed method is not restricted to the resin-phantom parameters used in the main text, but can be re-optimized for more realistic skull-like acoustic properties when these are incorporated into the design model.

To further evaluate robustness to model mismatch, we fixed the metasurface designed for the representative case with a skull sound speed of 2700 m/s and tested its performance under sound-speed variations from 2400 to 3000 m/s. Representative focal-plane pressure-amplitude distributions are shown in Figure S6(a-c), and the corresponding quantitative metrics are summarized in Figure S7. Across the tested range, the focal pattern remains qualitatively preserved. The mean spatial error remains on the order of sub-millimeter to approximately 1 mm, while the coefficient of variation (CV) of peak focal intensity remains below approximately 10%, with the best performance observed near the design point. These results indicate that, although the focusing quality is naturally optimized for the assumed acoustic properties and gradually changes as the parameter mismatch increases, the metasurface retains a meaningful degree of robustness to moderate sound-speed deviations.


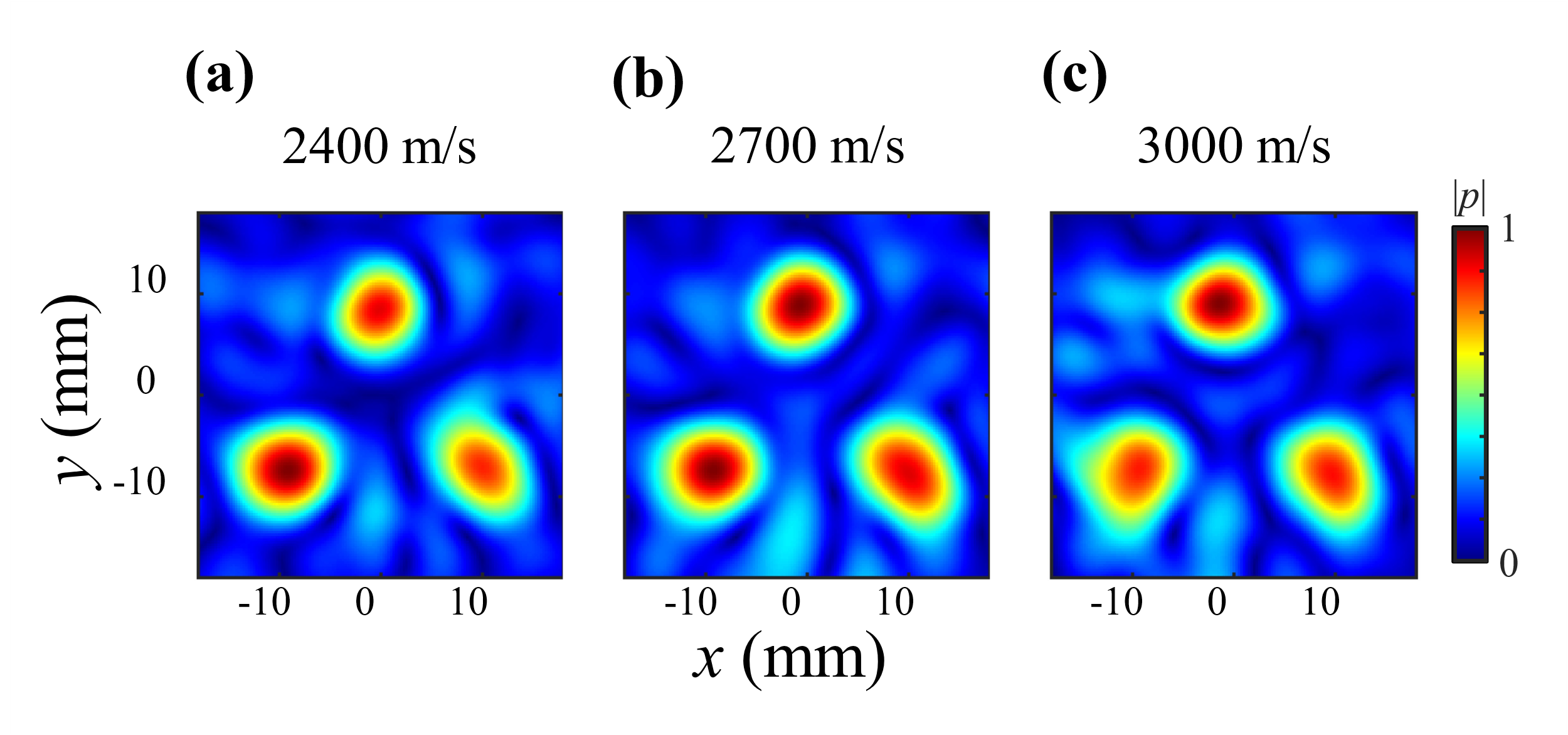


**Figure S6. Simulated sound pressure amplitude distributions using the same metasurface (designed for 2700 m/s), while through the skull with different sound speed (from 2400 m/s to 3000 m/s), illustrating robustness to skull sound-speed mismatch.**


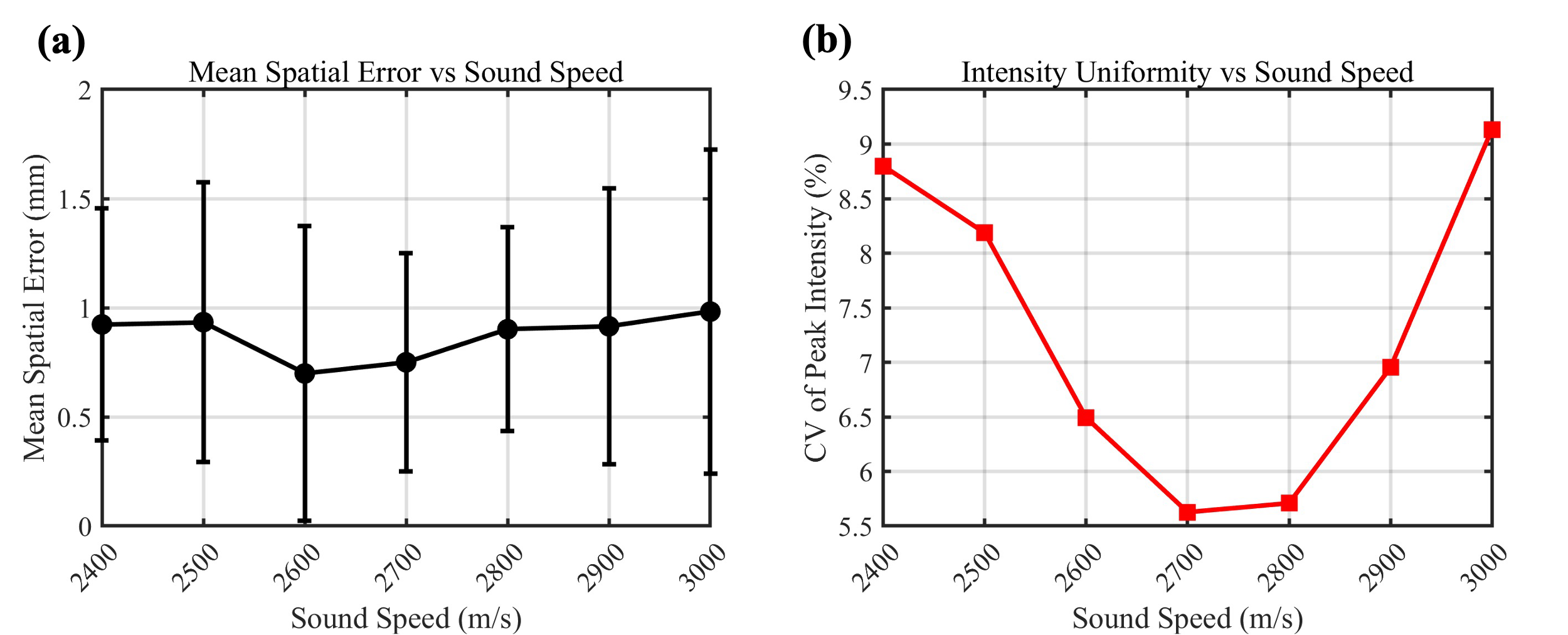


**Figure S7. Quantitative evaluation of robustness to skull sound-speed mismatch.** The metasurface was designed for 2700 m/s, and the skull sound speed varies from 2400 m/s to 3000 m/s. **(a)** Mean spatial error of the three focal spots as a function of skull sound speed. **(b)** Coefficient of variation (CV) of peak focal intensity as a function of skull sound speed.
